# “There is no one who helps you with it”: experiences of people with long COVID regarding medical care, therapeutic measures, and barriers in the German healthcare system: results of a qualitative study with four focus groups

**DOI:** 10.1186/s12913-023-10170-x

**Published:** 2023-10-26

**Authors:** Tim Schmachtenberg, Gloria Königs, Anita Dragaqina, Sascha Roder, Frank Müller, Christina Müllenmeister, Dominik Schröder, Alexandra Dopfer-Jablonka, Katharina Vieth, Iman El-Sayed

**Affiliations:** 1 Department of General Practice, University Medical Center Göttingen, Humboldtallee 38, 37073 Göttingen, Germany; 2 Department of Rheumatology and Immunology, Hannover Medical School, Carl-Neuberg-Str. 1, 30625 Hannover, Germany; 3 Department of Family Medicine, College of Human Medicine, Michigan State University, 15 Michigan St NE, Grand Rapids, MI 49503 USA; 4 German Center for Infection Research, Partner Site Hannover-Braunschweig, Feodor-Lynen- Str. 26, 30625 Hannover, Germany

**Keywords:** Long COVID, SARS-CoV-2, COVID-19, Medical care, Therapeutic interventions, Barriers to care, Healthcare system, Germany, Qualitative study, Focus groups

## Abstract

**Background:**

Many people experience long-term symptoms such as fatigue, cognitive problems, or shortness of breath after an acute infection with COVID-19. This emerging syndrome, known as long COVID, is new and complex in many aspects. This study aims to collect the experiences of people with long COVID with ambulatory healthcare structures.

**Methods:**

Four focus groups were conducted with a total of 23 adults with long COVID in June and July 2022. These discussions were audio-recorded, subsequently transcribed, and analyzed using the qualitative content analysis of Mayring and Kuckartz.

**Results:**

Fourteen out of 19 participants who had a primary care encounter regarding their long COVID symptoms did not perceive it as helpful. Many respondents reported that their general practitioners did not take their long COVID symptoms seriously and did not refer them to specialists or made therapeutic recommendations. However, some participants reported that they were prescribed non-pharmaceutical therapies (e.g., group meetings supported by psychotherapists, occupational therapy, etc.) that improved their condition. 14 of 23 respondents perceived care barriers such as providers’ lack of awareness of long COVID, poor access to specialists, a lack of specialized care (e.g., long COVID clinics), or high bureaucratic hurdles for specific healthcare services. To improve medical care, participants suggested campaigns to raise awareness of long COVID among healthcare providers and the general population, increase research and government investments regarding the development of treatment structures for long COVID, expanding existing therapeutic services, and establishing one-stop shops for integrated specialist healthcare for people with long COVID.

**Conclusions:**

Several implications for healthcare professionals and policymakers can be derived from this study: (1) general practitioners should take the symptoms of long COVID seriously, assume a care coordinating role, make referrals, and establish contact with long COVID clinics; (2) care planners should focus on developing interprofessional evidence-based care and treatment approaches for long COVID; (3) existing care structures such as long COVID outpatient clinics should be expanded. The overarching goal must be to develop consistent guidelines for long COVID diagnosis, care, and treatment.

**Trial registration:**

The study is registered in the German register for clinical trials (DRKS00026007, first registration on 09/09/2021).

**Supplementary Information:**

The online version contains supplementary material available at 10.1186/s12913-023-10170-x.

## Background

More than 760 million people have been infected with SARS-CoV-2 (severe acute respiratory syndrome coronavirus type 2) worldwide [1]. In Germany, approximately 38 million COVID-19 cases have been reported by health authorities since the start of the pandemic (as of March 21, 2023) [2]. The SARS-CoV-2 virus pandemic, which turned into a global health crisis with high mortality worldwide, has had a negative impact on a number of areas of health and social life, including a particularly vulnerable group of older adults [3]. The authors of a systematic review estimate that at least 7.5% of non-hospitalized adults experience long-term symptoms after acute infection with COVID-19 [4]. The prevalence is even higher in hospitalized patients [4, 5]. Terms such as *post-* and *long COVID-19* have been introduced to describe the phenomenon of persistent or new symptoms after acute COVID-19 infection. In the following, we use the definition of “long COVID” proposed by the German Federal Institute of Public Health Robert Koch-Institute (RKI). Accordingly, long COVID can be diagnosed when symptoms persist or are newly onset after an acute illness phase of SARS-CoV-2 infection of four weeks and cannot be explained by other conditions [6]. Long COVID is considered a multisystem disease with a heterogeneous clinical picture [7]: The most common symptoms are fatigue, cognitive problems, shortness of breath, headache, joint and muscle pain, and psychological problems (e.g., anxiety or depression) [7–13]. Long COVID can in general affect any person infected with SARS-CoV-2; however, female gender and hospitalization during the acute phase of infection are associated with an increased risk [8, 14–16]. Studies also suggest that there are age-specific differences in the course and duration of long COVID. Although no correlation between older age and an increased risk of long COVID has been shown to date [17], older adults (≥ 40 years) seem to suffer more frequently from symptoms persisting three months or more after the acute phase of COVID-19 (post COVID) than younger adults (< 40 years) [18]. Immunologic processes leading to persistent inflammation are likely causative of long COVID. Currently, no effective treatment targeting the causes of the disease is known [8, 19]. Although other protracted postinfectious syndromal courses can occur in other infectious diseases such as mononucleosis or measles [20–23], healthcare structures are struggling to provide care for large numbers of people with long COVID [24].

First studies have shown that many people with long COVID perceive their medical care as inadequate [25, 26]. They describe a lack of acceptance of their symptoms by health professionals, which leads to uncertainty and the feeling of being left alone [25, 27, 28]. This represents an obstacle to needs-based medical care.

This study uses a qualitative approach to provide insights into the experiences of people with long COVID regarding ambulatory medical care, drug or non-drug therapies, and access barriers to services relevant to affected individuals in the German healthcare system. The paper complements the existing literature with a multidimensional view of current challenges in the care of people with long COVID from the perspective of those affected. Problem analyses are conducted on the micro (interaction with physicians), meso (access barriers at certain care facilities), and macro (structural barriers in the healthcare system) levels. Furthermore, examples of care and positive experiences of participants with therapeutic measures are used to identify starting points for improving the care situation of people with long COVID. The purpose of this work is to show care planners and providers how people with long COVID experience their healthcare situation, at what points in the healthcare system they perceive gaps and access barriers regarding formal services, and what interventions to address existing healthcare problems they would like to see from policymakers.

## Methods

The study is part of the multicenter research project “DEFEnse Against COVID-19 STudy - Looking forward” (DEFEAT Corona) [29], which examines the phenomenon of long COVID from both biomedical and social science perspectives. In addition to a longitudinal cohort study of people with long COVID, one-on-one interviews on social participation have been carried out. The results of these interviews have already been published in a peer reviewed journal [30].

In this subproject, four focus groups were conducted with a total of 23 adults with long COVID. The focus groups were developed specifically for this study and have not been published elsewhere. An English version of the moderation guideline for the focus groups is presented as a supplementary file. This method was chosen because focus groups have a higher performance than individuals due to the collective knowledge base [31]. High performance should be particularly helpful in identifying potential care barriers and approaches to improve the care situation, which are the main objectives of this research. The study lasted approximately six months. Study results are reported using the consolidated criteria for reporting qualitative research (COREQ) [32].

### Inclusion and exclusion criteria

Eligibility criteria for participation in the focus groups were having long COVID according to the RKI definition (symptoms persisting beyond the acute phase of SARS-CoV-2 infection of four weeks and not explained by another diagnosis) at the time of recruitment in June 2022, a minimum age of 18 years, and consent to participate in a face-to-face discussion round. Individuals whose cognitive or physical impairment was too severe to take part in a discussion group or travel to the location where the data were collected were not included for pragmatic and research ethics reasons.

### Recruitment

A convenience sample was recruited for this study. The recruitment strategy included a call for participation (a) on the websites of the Department of General Practice at the University Medical Center Göttingen (UMG) and the Hannover Medical School (MHH) and (b) on the social media channels (Instagram) of the two university hospitals. Furthermore, (c) respondents from individual interviews conducted on social participation suggested potential participants for the focus groups (snowball system). The call for participation targeted adults affected by long or post-COVID with self-perceived impaired health status and reduced quality of life. Recruitment efforts occurred in May and June 2022. Prior to the focus group meetings, interested individuals were contacted by phone or email to verify inclusion and exclusion criteria and to inform them about the focus group procedure. Additionally, the moderators introduced themselves and briefly explained the study rationale. Furthermore, written informed consent to participate was obtained. The sample size was determined following common guidelines for conducting focus groups, which consider three to five focus groups [31] with five to eight participants each [31, 33–35] to be sufficient to make assumptions on a study subject. Since smaller groups are appropriate for complex topics [33], the focus groups were planned with seven participants each. The 28 participants were randomly selected from a total of 35 interested individuals who met the inclusion criteria. The final number of 23 participants resulted due to five participants no-showed at the focus group appointment despite prior agreements. Unaware to the investigators, a few participants were reportedly familiar with each other prior to data collection. The participants received a compensation of 40 Euros (approx. 40 USD) for their participation.

### Development of moderation guideline

The moderation guideline for the focus groups was based on methodological literature [31, 33, 34] and the findings of previously conducted one-on-one interviews on social participation with people with long COVID [30]. The discussion topics were defined and the moderators’ input was kept brief and open-ended [31, 33–35]. The guideline was developed by three researchers (TS, SR, and IES) in a discursive process on June 10, 2022. Several guiding themes emerged from which the following questions relevant to this study could be derived: (1) What are the experiences of people with long COVID with ambulatory medical care in the context of this disease? (2) To what extent do they use therapeutic interventions and to what extent do they experience them as helpful? (3) How do people with long COVID perceive the care situation and to what extent do they face obstacles in accessing services of the healthcare system? (4) What are the hopes and expectations of people with long COVID regarding the future provision of care? To provide a stimulus for discussion, a short radio feature about long COVID in everyday life was played at the beginning of the focus groups [36]. To illustrate questions, the researchers included quotes from one-on-one interviews with people with long COVID previously conducted by one of the authors (SR) within the project DEFEAT Corona as a further stimulus. The moderation guideline provided as a supplementary file was not shared with the participants.

### Data collection

Data collection was conducted in two sessions on June 22 and July 6, 2022. The focus groups were held in seminar rooms at the University Medical Center Göttingen and the Hannover Medical School. Participants and moderators were seated at rectangular tables with wrap-around seating. This face-to-face setting was chosen to facilitate the emergence of group dynamic processes and interaction between the participants. Conducting the focus groups in presence was chosen to get a better situational understanding of the participants and their interaction [37]. To reduce the risk of infection, protective measures mandated by the university hospitals at the time of the data collection were followed, such as checking a daily negative SARS-CoV-2 rapid antigen test result from a testing center, maintaining the minimum distance between participants, and wearing an FFP2 mask. No persons other than the participants and researchers were present in the focus groups. All focus groups were conducted by three researchers (TS, SR, and IES) in alternating roles, with each person moderating, co-moderating, and taking minutes at least once. The focus groups were recorded using two digital audio recording devices. Before the discussion started, sociodemographic information was collected. In addition, the researchers noted the speaking order and conspicuities during the discussions. The focus groups lasted between 60 and 80 min (mean: 69 min).

### Data evaluation

In the first step, the audio recordings of the focus groups were transcribed in terms of content-semantics following Dresing/Pehl and Kuckartz [38, 39] and the transcripts were checked for accuracy. Afterward, the data was evaluated using qualitative content analysis of Mayring and Kuckartz [40, 41]. The study team, which consisted of a medical sociologist (TS), a social pedagogue (IES), and two study assistants (GK and AD), developed a category system in an iterative and discursive process. A combination of deductive and inductive category development was used. First, the main categories were deductively derived from the moderation guideline. Then, further categories were generated inductively in an open coding process. This was followed by axial coding of the subcategories. The content analytic process was repeatedly discussed and a codebook was developed with the definitions of the categories, example citations, and semantic meanings. Subsequently, the final codings were conducted and paraphrases and generalizations were derived. Table 1 provides an overview of the generated categories. In total, the category system consists of eleven main categories, 25 top categories, and eight subcategories. This work focuses on the main category orientation in the healthcare system including four of its top categories and one of its subcategories as well as the main category suggestions for supportive measures (highlighted in the table). In the final analysis step, all generalizations were tabulated, systematically compared, and interpreted. MAXQDA software version 20.0.8 (VERBI Software GmbH, Berlin, Germany) was used to analyze all data. Data evaluation focused exclusively on the long COVID complaints of the participants; no comorbidities were analyzed.

**Table 1:** Category system

Main categories	Top categories	Subcategories
1. Personal information		
	1.1. Medical history	
	1.2. Attitude to life	
2. COVID-19 infection		
	2.1. Consequences of COVID-19 infection	
		2.1.1. Complaints during the acute phase
		2.1.2. Persistent limitations
		2.1.3. New complaints
	2.2. Improvement of the complaints	
	2.3. Emotional state and expectations regarding the course of the disease	
	2.4. Coping with complaints	
3. Information procurement		
4. Measures for self-protection and protection of others		
**5. Orientation in the healthcare system**		
	5.1. **Criticism of the healthcare system**	
		5.1.1. **Assessment of medical ability to act**
	5.2. **Positive experiences with medical care**	
	5.3. **Negative experiences with medical care**	
	5.4. Diagnostic measures	
	5.5. **Therapeutic measures**	
		5.5.1. Rehabilitation experiences
	5.6. COVID-19 vaccination	
		5.6.1. Side effects of COVID-19 vaccination
6. Effects on the own life situation		
	6.1. Consequences for everyday life	
		6.1.1. Effects on personal interests
		6.1.2. Effects on social participation
7. Social environment		
	7.1. (In-)Visibility of the disease	
	7.2. Support and consideration by the close social environment	
	7.3. Support and consideration by the broader social environment	
	7.4. Effects on the social environment	
8. Criticism of society		
	8.1. Own social role perception	
	8.2. Evaluation of government measures	
9. Occupational activity		
	9.1. Support and consideration by the working environment	
	9.2. Effects on the occupation	
	9.3. Occupational perspective	
10. Reactions of the focus group participants		
	10.1. Reactions of the focus group to the stimulus	
	10.2. Reaction to other focus group participants	
	10.3. Feedback to the moderators	
**11. Suggestions for supportive measures**		

Categories analyzed for this study are highlighted in bold

### German healthcare system

The German healthcare system is a social insurance system in which health insurance funds largely finance medical care. For this, companies and employees pay into the health insurance funds. All citizens residing in Germany are required to take out health insurance coverage. While most people and especially employees are compulsorily insured in statutory health insurance, some groups of people such as civil servants or the self-employed can also choose private health insurance. People with statutory health insurance are entitled to free treatment. GPs act as coordinators of medical care, but patients can also consult specialists independently [42]. With specialists, there are often long waiting times [43]. Private and public providers operate side by side in the German healthcare system [42]. Rehabilitation measures such as outpatient or inpatient medical rehabilitation, vocational rehabilitation, or rehabilitation for post- or long COVID are not paid for by health insurance, but by pension insurance. However, applications must be written and approved for this [44].

## Results

The main statements of the participants presented in this chapter are substantiated with original quotes from the focus groups. Discussions were forward translated into English. “F” at the end of the quotes and in the tables stands for focus group and “P” for participants.

### Participant cohort

39 individuals responded to recruitment efforts, of which 35 met the inclusion criteria. From these 35 individuals, 28 were randomly selected and invited to the focus group. Three participants were not present on the day of the data collection without prior cancellation. Two other individuals canceled on short notice, one of them due to health problems. A total of 23 participants eventually attended the focus groups.

The participants were between 19 and 63 years old (mean: 41.6 years), 16 were female (70%), and seven male (30%). With the exception of one female student, one male student, and one person who received a disability pension, all participants were employed. Table 2 shows the selected socio-demographic characteristics of the participants (sorted chronologically according to the focus group dates and the order of speakers).

**Table 2:** Overview of sociodemographic characteristics of participants

Focus group	Participant	Sex	Age	Occupation	Interview date (setting)
**F1**	**P1**	Male	48–57 years	Banker	06/22/2022 (Göttingen)
	**P2**	Male	48–57 years	Hygiene manager	06/22/2022 (Göttingen)
	**P3**	Female	48–57 years	Nurse	06/22/2022 (Göttingen)
	**P4**	Female	38–47 years	Lab technician	06/22/2022 (Göttingen)
	**P5**	Male	18–27 years	College student	06/22/2022 (Göttingen)
**F2**	**P1**	Female	No information	Office clerk	06/22/2022 (Göttingen)
	**P2**	Female	18–27 years	Preschool teacher and student	06/22/2022 (Göttingen)
	**P3**	Female	18–27 years	Lab technician	06/22/2022 (Göttingen)
	**P4**	Female	18–27 years	Student	06/22/2022 (Göttingen)
	**P5**	Female	58–67 years	Board member of a company	06/22/2022 (Göttingen)
**F3**	**P1**	Female	28–37 years	Social worker	07/06/2022 (Hannover)
	**P2**	Female	48–57 years	Journalist	07/06/2022 (Hannover)
	**P3**	Male	48–57 years	Cook	07/06/2022 (Hannover)
	**P4**	Female	48–57 years	Medical assistant	07/06/2022 (Hannover)
	**P5**	Female	38–47 years	Employee in geriatric care	07/06/2022 (Hannover)
	**P6**	Male	58–67 years	Driver	07/06/2022 (Hannover)
**F4**	**P1**	Male	48–57 years	Engineer	07/06/2022 (Hannover)
	**P2**	Female	28–37 years	Beautician	07/06/2022 (Hannover)
	**P3**	Female	48–57 years	Teacher	07/06/2022 (Hannover)
	**P4**	Male	48–57 years	Police medical inspector	07/06/2022 (Hannover)
	**P5**	Female	28–37 years	Paramedic	07/06/2022 (Hannover)
	**P6**	Female	28–37 years	Preschool teacher	07/06/2022 (Hannover)
	**P7**	Female	28–37 years	Disability beneficiary (former office manager)	07/06/2022 (Hannover)

Analysis of the content presented identified themes in five categories: (1) positive and (2) negative experiences with medical care, (3) experience of therapies, (4) perceptions of challenges and barriers to seeking services in the German healthcare system, and (5) suggestions for supportive measures.

Table 3 shows the complaint profile of the participants at the time of the focus group, current and past care contacts with physicians and psychologists, and current and past therapeutic measures. Six participants had solely encountered their general practitioners (GP) for their long COVID complaints, two participants reported only consulting specialists, and eleven participants visited both GPs and specialists. One person stated that he had not sought medical attention due to long COVID-related health problems. Three persons did not provide any information in this regard. In total, 19 of the 23 participants had personal experiences with ambulatory medical providers for their long COVID complaints.

**Table 3:** Overview of reported (long COVID) complaints, (physician) care settings, and (implemented) therapeutic measures

Participants	Reported complaints	Medical care (by)	Therapeutic measures
**F1 – P1**	Reduced resilience, erectile dysfunction, depressed mood, sleep disturbances, personality change	GP, pulmonologist, cardiologist	Psychosomatic rehabilitation
**F1 – P2**	-^*^	GP, pulmonologist, gastroenterologist	Physiotherapy, respiratory therapy, build-up of intestinal flora, physical activity (walks, cycling), regeneration (rest periods).
**F1 – P3**	Shortness of breath, reduced endurance, water retention in the legs	Pulmonologist, cardiologist	Rehabilitation
**F1 – P4**	Impaired lung function	-^*^	Respiratory therapy
**F1 – P5**	Impaired lung function	GP, pulmonologist	-^*^
**F2 – P1**	Fatigue, dizziness, headache, concentration problems	GP, pulmonologist	-^*^
**F2 – P2**	Nerve pain, insensitivity (in the legs), hair loss	GP, cardiologist	Drug treatment (ibuprofen)
**F2 – P3**	Concentration problems, memory problems, reduced physical performance	No medical consultation	-^*^
**F2 – P4**	Heart problems, hair loss	GP, pneumologist, internist	Drug treatment (“asthma spray”), physiotherapy, rehabilitation sports
**F2 – P5**	Reduced physical performance	GP	-^*^
**F3 – P1**	Fatigue, depressed mood	GP	-^*^
**F3 – P2**	Fatigue, shortness of breath, muscle pain, concentration problems	-^*^	-^*^
**F3 – P3**	Pulmonary problems, nerve pain, loss of energy	GP, cardiologist, pulmonologist, sports doctor	Therapeutically guided fitness training, respiratory training, rehabilitation (pulmonary training, sports therapy), intake of vitamins, drug treatment (temporary intake of painkillers, drugs to regulate the heart rate)
**F3 – P4**	Fatigue, rheumatic complaints, concentration problems	GP, rheumatologist	Drug treatment (cortisone)
**F3 – P5**	Loss of energy, depressed mood	GP, cardiologist, psychologist	Rehabilitation
**F3 – P6**	Fatigue, impaired sense of smell	GP, orthopedist, rheumatologist, pulmonologist	Respiratory training, rehabilitation, training-therapeutic rehabilitation aftercare
**F4 – P1**	Fatigue, headache, concentration problems, memory disorders	GP	Occupational therapy, physiotherapy, rehabilitation
**F4 – P2**	-^*^	GP, physician from a coagulation outpatient clinic	Group meetings supported by psychotherapists, neurofeedback
**F4 – P3**	Fatigue, muscle pain, nausea, increase in pulse, concentration problems, word-finding disorders, impaired memory	GP	-^*^
**F4 – P4**	Impairment of short-term memory, mood swings	GP	Group meetings supported by psychotherapists, neurofeedback
**F4 – P5**	Fatigue, headache, histaminosis	-^*^	Occupational therapy, physical therapy, sports therapy, cognitive performance training, outpatient rehabilitation
**F4 – P6**	-^*^	GP	-^*^
**F4 – P7**	Noise sensitivity	Physician from rehabilitation medicine	Occupational therapy, physiotherapy

* = No information provided

### Positive experiences with medical care

A total of eight participants perceived that their GPs took their long COVID complaints seriously. They experienced that their GPs listened, took interest in their situation, and addressed their problems. *“[My GP] definitely takes it seriously” (F2, P1). “My GP was interested there” (F1, P1).* Five participants reported receiving further support from their GPs. For five participants, this was in the form of referrals to specialists (internal medicine, cardiology, neurology, pulmonology). *“My GP […] referred me to the [pulmonologist]. There I was also helped a little bit” (F2, P4). “My doctor […] sent me straight to the cardiologist” (F3, P3).* Two participants each received therapy recommendations and further information about long COVID from their GPs. In particular, the initiative and proactive communication of individual GPs gave the participants a good feeling. *“Thank God I have a GP who also has little experience in this area, but who supports me 100%. She […] calls you and does and does. I’m lucky to have here.“ (F4, P1)*.

One participant expressed complete satisfaction with both the support provided by his GP and the extensive specialist examinations. *“I’m completely examined, by the pulmonary specialist, by the sports doctor. Then I just get tested to see how my performance is” (F3, P3).* The immediate acceptance of his complaints by the treating GPs and specialists, their sensitivity to long COVID signs, and the timely examinations and treatment efforts have provided confidence and security for the participant. *“All the doctors I’ve been to have taken me seriously right away and said ‘no, those are the signs of long or post COVID.‘ That’s when I was lucky that my GP did a lot, tried a lot” (F3, P3).*

### Negative experiences with medical care

While the relation between positive and negative experiences was relatively balanced regarding the acceptance of participants’ long COVID complaints by GPs (seven people felt that they were not taken seriously in this regard), the negative experiences predominated in the other aspects of medical care discussed. Several respondents described feeling that their GPs did not emphasize their situation and could not comprehend their perception of their complaints, which eventually even worsened their well-being.

*“I then hear from my doctor, so it came across as ‘pull yourself together a bit and go for a bike ride every now and then and start doing sport and then it will be fine’. And when I told him about this crash and he told me what he understood by a crash, I left the practice, and tears came to my eyes again. And I thought ‘he doesn’t know how I feel at all. He doesn’t know what’s happening to my body at all’. I also felt so lost and so alone because the understanding he has of the disease I have is completely different than what I experience.“ (F4, P3)*.

Eight participants reported that they received no supportive gestures or understanding at all from their GPs. They received neither advice on treatment options, possible next diagnostic steps, and measures for handling the complaints, nor advice on how to cope in their situations with their everyday lives. Several participants pointed out that their GPs advised them to wait and be patient, as the symptoms would go away on their own. *“And that was also the tenor again for three months, that she [the GP] said ‘wait and see’” (F3, P1).* Two respondents experienced a reduction of their complaints to psychological or psychosomatic symptom patterns and a lack of acceptance of their suffering due to their young age. *“And then there are doctors who label you directly as psychosomatic […] and say ‘You are such a young woman’” (F4, P7).* The lack of (external) visibility of the condition was perceived as a possible obstacle to the recognition and acceptance by their physicians. *“A doctor once said to me: ‘I can’t see your [condition] at all’” (F4, P7).*

Due to the lack of acceptance by physicians, participants conducted extensive research to build up disease-management skills and to compensate (at least partially) for the lack of professional support. Many participants stated that they had made great efforts to convince their GPs that their symptoms required treatment. Some participants reported experiencing rejection from their GPs even when they came to the practice well-prepared, brought examination and lab results indicating a health problem, and made treatment suggestions. *“You already work everything out so that you can actually evaluate it medically well. He [the GP] took a quick look at my first [blood] test results [with high inflammation values] […] and that was it. For him, that was ‘please don’t use the C-word [Corona]’” (F4, P4).* Some respondents experienced that their GPs advised them against certain therapeutic measures such as rehabilitation or consulting specialists for further diagnostics. In some cases, this led to delayed care.

*“When he [the GP] got the report from the pulmonary specialist, […] he said to me ‘Physiotherapy? Do you think you need that? You’re doing sports. Rehabilitation? I’m not gonna do the paperwork for your rehabilitation. And then I say ‘Why? ‘I don’t think we’re there yet’. […] ‘Well, I wouldn’t do all this rehabilitation stress.‘” (F1, P2)*.

*“I hadn’t consulted any specialists yet because my GP didn’t think I needed them.“ (F2, P2)*.

Overall, 14 participants concluded that consultation with GPs and specialists was not helpful in the context of their long COVID symptoms. Consequently, many respondents felt they had been left alone and that they had to cope with their situation without any support from their GPs. *“There is no one to help you with it. You have to do everything yourself” (F3, P6).* The experiences of rejection, in combination with the reduced energy resources due to fatigue, led several participants to stop contacting their GPs with their long COVID complaints. *“My GP, I don’t need to go at all, I only go because I need a sick note further. He says bluntly ‘I can’t help you’” (F2, P2).* Furthermore, some individuals with long COVID lost trust in the overall German healthcare system and the service they provide.

*“The trust, the credibility to even go there or […] energy, I need that for myself at the moment. But by having to work things out or what else can you think of, where can you take him by the hand again? Your doctor. That’s actually where we are right now in this situation. […] When you have these experiences, you really wonder about Germany, 21st century, medicine? What other countries want to have so much, where we are. If we really knock and say ‘hello, help me’. From a basic point of view, not really” (F4, P4).*

### Experience of therapies

Participants mentioned several therapeutic interventions that they perceived as helpful in terms of their symptoms, well-being, and coping with daily life activities. Five respondents were satisfied with their specialist therapies. Three participants reported that they experienced occupational therapy and physical therapy treatments as helpful to maintain physical and cognitive abilities. *“I’ve done quite a bit of occupational and physical therapy tailored just to me. That already does a lot for me to stay in shape as much as I can. If I wouldn’t have that.“ (F4, P1).* Additionally, three participants found it supportive to take part in group meetings supported by psychotherapists, in which they could share their experiences of handling long COVID with other patients. Participants described these groups as emotionally uplifting, but also helpful for the recommendations that were shared for alleviating symptoms (e.g., by adjusting diet, exercise, and sleep habits) and managing everyday activities.

*“The coping group, […] that I get there with tips with diet, exercise, sleep and everything once a week. […] This weekly exchange alone. We are there nine in the group, also once there to know ‘what have you already done?‘, ‘what has helped you there something?‘. The psychotherapist, […] that she then directs us a little bit correctly. […] That gave us a lot, built us up. […] You can try out a lot of things that you wouldn’t have thought of yourself. That has been very helpful” (F4, P4).*

Individual participants perceived cognitive training in a neuropsychological setting, neurofeedback therapy, respiratory training, training-therapeutic rehabilitation aftercare (T-RENA), and physician-guided vitamin therapy as helpful. *“I’ve been doing regular exercise since November, first done in the cure, and was able to continue this fitness training, which is called T-RENA. That goes over the pension fund […]. There I go twice a week and do sport there. And it’s good for me […]. I do everything, cycling, so hand cycling 10 minutes” (F3, P6). “A lot with vitamins, not necessarily painkillers” (F3, P3).*

One respondent also reported that, as part of a sports study, he received individual training plans and a heart rate watch with an associated app for monitoring his heart rate. In this way, his thresholds of overexertion were determined and he was able to adjust his activities accordingly. The watch provided the participant with immediate feedback regarding the degree of stress of certain activities and alerted him by an acoustic signal if his heart rate exceeded a limit. *“My heart rate must be no more than 115, I can’t go over, because otherwise, it’s too stressful. There’s climbing stairs, ten minutes, there’s walking for half an hour, […] cycling for half an hour, but very slowly, and as soon as I’m over it, the watch beeps (F3, P3).* The data stored in the app was regularly evaluated and the participant’s activities were adjusted based on this data and the results of specialist examinations and performance tests. Furthermore, the respondent participated in a sports group to which he was referred by providers at a specialized long COVID outpatient clinic. *“Yes, I had been here on this big examination day [of the long COVID outpatient clinic of the Hannover Medical School] and the colleague says, there is also a sports group here and whether that would be something for me. Then I went straight there” (F3, P3).* As a result of utilizing these complementary medicine therapies, the patient experienced relief from his complaints and an improvement in his well-being.

In contrast, four participants were not satisfied with the therapeutic measures prescribed by their physicians. Two participants reported receiving drug treatment. While one person received an asthma spray, one respondent was prescribed a painkiller. *“I was told ‘there is nothing. Take an ibuprofen.‘ But I can’t take ibuprofen every day either. That goes on my stomach at some point” (F2, P2).* For two participants, the physician’s recommendations were limited to rest and exercise, leaving them perplexed and in despair. *“’What should I do?‘ ‘Head thing and rest and go for a walk and ride a bike’” (F1, P2).*

### Perceptions of challenges and barriers to seeking services in the German healthcare system

14 of the 23 respondents criticized certain aspects, structures, or institutions in the German healthcare system. Ten participants noted a general lack of knowledge, treatment options, and too few approaches to develop such care concepts. According to the participants, lack of knowledge severely limited the ability of GPs and specialists to act. *“There are no tools that doctors can take in their hands and say ‘this is what I’m giving you and this is what I’m helping you with’” (F3, P2).* Five participants criticized the waiting times of several months for specialist appointments such as pulmonologists or cardiologists and saw this as an access barrier to receive the care they need. *“With the cardiologist, you call ‘come in half a year’. […] I find that alarming in Germany. […] If you really have the demand and then call somewhere and they say ‘in half a year’, the next one says ‘this year it will be nothing at all’” (F3, P3).*

Four respondents referred to a lack of specialized long COVID clinics. Two of them perceived the long wait times for long COVID consultations as impairing their care and recovery. *“I reached out [to two] […] long COVID outpatient clinics […]. […] There was stalling […], I say ‘When am I going to get an appointment?‘ ‘You’ll have to wait another year for that’. I say, ‘When I’m dead, I don’t need an appointment anymore’” (F3, P6).* Three respondents perceived bureaucratic hurdles (e.g., clinics asked for test reports in advance, long processing times for rehabilitation applications) and lack of contact persons (at authorities and providers) as access barriers to care services such as rehabilitation measures and long COVID consultations. *“I noticed it with rehabilitation, the applications you have to fill out, it’s immense. Getting that application in the first place. […] That also took forever and you also have questions about what you have to fill out. There, too, you didn’t have a contact person. It was just so much bureaucracy […]. And I think many people shy away from that” (F3, P3).*

Three participants criticized the design and poor information transfer regarding the preparation, conduct, and follow-up of scientific studies in the field of health research. As a result, these individuals felt that, as affected persons, they often did not benefit from the potential added values of such studies, or only to a limited extent.

*“The information flow [in studies] works poorly” (F3, P6). “It was about rheumatism […]. Then I had filled out all the questions and then I think it said at the beginning, at the bottom, I could then make an appointment, because somehow with a rheumatism practice […] is cooperated, but it came somehow not. […] I just couldn’t specify an appointment anywhere, I thought that was a pity. And then I would have had to fill out all the questions again. That is somehow a bit strangely presented in any case. […] I only became aware of the group here through a friend” (F3, P5).*

Respondents with inpatient or day-care rehabilitation experience saw a further problem in the lack of orientation of rehabilitation clinics and programs toward long COVID. In this context, one person used an ironic remark to criticize the lack of flexibility of the German Pension Insurance concerning timely adaptation to crisis situations such as pandemics.

*“At the point [treatment of long COVID], in my eyes, the rehabilitation structure is not set up for that either. If I do not have just quite clearly pneumatological problems at the lung or something, then I am sorted by default and […] treated psychosomatically, for example […]. I don’t expect that from the pension insurance, flexibility and temporal speed, to adjust to things within two years, but I believe that there would also be a need for it.“ (F1, P1)*.

### Suggestions for supportive measures

The participants not only shared their experiences with the relevant healthcare institutions and actors but also discussed possible approaches to solving the described healthcare problems. Seven respondents expressed the wish that one-stop shops in the health sector and the population be specifically sensitized to the issue of long COVID through measures such as information campaigns by the federal government. This should include a focus on confronting the common misconceptions in parts of society and the healthcare system that persistent COVID complaints have psychological causes or primarily affect older adults.

*“I would like to see maybe a campaign like that from the federal government about it [long COVID]” (F1, P5). “I also find the idea with this campaign very good and what I would also find important would be that people are also told plainly and clearly, because what you really only hear is ‘Are you sure that you really still have organic problems and that this is not only the psyche?‘ […] Of course, at some point, it goes to the psyche, but there are organic problems (F1, P4).“*

Five participants emphasized the need to increase research activities and government investment regarding the treatment of long COVID. *“That maybe this [hyperbaric oxygen therapy] can be offered in a setting of higher-paying trials, that more is tried” (F4, P5).* Likewise, five individuals wished for an expansion of existing therapeutic services such as rehabilitation sports or group therapy. In addition, participants clearly stated that access to support services and studies should be made easier. *“That also, for example, physiotherapy prescriptions are not limited due to any budget things and that also everyone has access” (F4, P7).* Moreover, participants highlighted the need for better networking and communication between the treating physicians. To enable integrated healthcare, the respondents recommended the establishment of one-stop shops where the results of different specialist examinations are brought together by a medical specialist acting as a fixed contact person. The participants highlighted the concept of the long COVID outpatient clinic of the Hannover Medical School and the establishment of specialist centers focusing on long COVID.

*“The way it is at the Hannover Medical School. That you have your GP at home and he controls it, all the examination things […], but then you say, I can go to the cardiologist tomorrow or whatever, and that you have the one contact person.“ (F3, P3)*.

*“That would be the smart thing, if you had such a center, really integrative and the psychologist is there, the rheumatologist is there and you could do everything in this center. And there are also people who know about this disease and seriously deal with it and also combine their knowledge.“ (F3, P2)*.

## Discussion

### Summary experiences with medical care

The analysis of the four focus groups with 23 participants with long COVID from Germany showed that the majority of participants had negative experiences with ambulatory care. Many participants experienced that their complaints were not taken seriously by GPs, who mostly serve as the first point of contact for patients in the German healthcare system and have a care coordinating function [42]. Furthermore, many respondents reported receiving no support in the form of medical prescriptions from their GPs predominated. In the German S1 guideline “Long/ Post-COVID”, GPs are initially recommended to adopt a wait-and-see approach in the case of clinical stability of symptoms after a basic diagnosis. According to the guideline, in-depth diagnostics and/or referral to appropriate specialists should only be offered if there are warning signs in the basic diagnostics and clinical deterioration [45]. Nevertheless, patients feel unappreciated if they are refused referrals or tests without explanation. 14 of the 19 participants who had a consultation with a GP or specialist due to long COVID did not find this consultation helpful. As a result, several respondents concluded that they would no longer contact their GPs regarding their long COVID complaints.

### Comparison with empirical studies and theoretical literature

Other studies also indicate that medical care is perceived as inadequate by many people with long COVID complaints. This represents an additional psychological burden for those affected [25, 26]. In a study by Wurz et al. [46], the majority of participants with long COVID reported ignorance or rejection by their primary care providers, leading to helplessness and frustration. Other studies emphasized the need to take patients seriously with their symptoms [27, 28]. At this point, some similarities with the experiences of people with Myalgic Encephalomyelitis and Chronic Fatigue Syndrome (ME/CFS) become apparent. Several studies have found that affected patients are mostly dissatisfied with the medical care and support they receive from the physicians they most frequently consult for ME/CFS (mostly GPs). Similar to many participants in this study, they are medically underserved [47, 48]. This led many patients with ME/CFS to utilize self-help services and alternative medicine [47]. In an interview study, GPs also reported major challenges in treating patients with long COVID. In particular, the diagnosis of long COVID, the handling of psychosocial problems and the ability to work of affected patients, and the prescription of rehabilitation measures caused great difficulties for GPs due to the lack of uniform guidelines [49].

Based on the interaction sociology of Goffman [50, 51], phenomena such as ignorance or rejection of patients with long COVID by health professionals and the absence of affected persons from the *stages* of medical care can be interpreted as *defensive and protective maneuvers* to maintain roles and social identities. Physicians are confronted by patients with long COVID with the fact that they have little expertise and treatment skills in a medical field. As a result, they cannot meet their own expectations and the expectations of their *audience* (patients, colleagues) regarding their professional medical role (providing sound medical advice to patients regarding their complaints). Consequently, interaction with individuals with long COVID poses a threat to the maintenance of the *social facade* (general conception of how to play a role) of the physician’s performance, which in turn is essential for the audience to perceive it as credible. To save their performance and avoid role conflict, some physicians ignore the complaints of patients with long COVID, thereby limiting their access to the medical practice stage. Vice versa, some individuals with long COVID also react to this double stigmatization (long COVID, endangering their own social identity as a physician) by protecting themselves from further attacks on the credibility of their performance as patients with legitimate complaints and not entering the stage (anymore) [50, 51]. As a consequence, affected people question their *everyday knowledge*, which is important for their orientation in their *life world*, regarding the consultation of experts for certain problems (physicians for health complaints) [51], which increases existing insecurities. Moreover, it can be assumed that patients with a relationship to their GP, which was previously characterized by reciprocal trust, are at risk of breaking off an *axis of resonance* (connection to other people), which is particularly important for people with chronic illnesses [52]. Some participants described an *experience of alienation* by pointing out that they felt left alone by healthcare providers. They sent (support) requests to the part of the social world relevant in the context of their complaints, and this did not answer them, which further increased the longing for resonance in the form of other people responding to their own life situation [52].

### Key findings experience of therapies

Several participants from this sample experienced positive changes in their health and everyday life situation through various non-drug therapies. Interventions perceived as helpful included group meetings supported by psychotherapists, individualized occupational therapy, physical therapy, training-therapeutic rehabilitation aftercare, respiratory training, computer-assisted cognition training, and app-assisted stress management. Quantitative studies should be conducted to examine the potential benefits of such therapies for a larger sample of people with similar symptoms. A lack of controlled studies on the prevention and treatment of long COVID is also pointed out by the Robert Koch-Institute [53]. For some respondents, reduction to single aspects of the disease led to dissatisfaction regarding therapeutic interventions. The participants perceived a lack of therapeutic concepts that address different complaints of people with long COVID. These experiences confirm current care guidelines that recommend multimodal treatment concepts and multi-professional collaboration among caregivers for long COVID [45, 54].

### Framing by existing evidence

Wurz et al. point out that many people with long COVID have difficulties in seeking treatments that improve their health situation. However, individual participants noted modest relief of symptoms following certain pharmaceutical and non-pharmaceutical treatments (e.g., dietary changes, physical therapy) [46]. Similar to the respondents from this sample, other studies also indicate that group meetings supported by psychotherapists, tailored training programs with strength and endurance exercises, and adapted rehabilitation measures have a positive influence on the recovery process of affected individuals [7, 55–57]. Davies et al. [58] emphasize that close follow-up with people with long COVID during the rehabilitation process and education about health conditions are essential. Several studies highlight the need for further research on the effectiveness of rehabilitation programs on the symptoms of people with long COVID [59, 60].

### Main results perceptions of challenges and barriers to seeking services in the German healthcare system

14 of the 23 participants in this study criticized certain aspects of the German healthcare system. Structural barriers to adequate (standard) healthcare for people with long COVID were identified as (1) lack of fundamental care concepts, (2) lack of awareness of long COVID in the healthcare system, (3) poor access to relevant specialists, (4) lack of specialized one-stop-shops such as specialized long COVID outpatient clinics, (5) high bureaucratic hurdles to arrange appointments or receive rehabilitation measures, (6) lack of specialization of rehabilitation clinics and programs. The few studies that consider the issue of healthcare barriers in long COVID briefly address individual problems reported in this paper, such as the lack of sensitivity of care providers to the concerns of affected individuals, the lack of scientific evidence and care concepts, and the lack of specialization of healthcare and service providers [9, 26]. Studies with people with ME/CFS also report barriers to accessing medical care, particularly specialist care, such as lack of financial (including insurance) resources, lack of knowledge about service availability, and geographic or logistical barriers [48, 61].

### Overview suggestions for supportive measures

The respondents made several recommendations for improving care for people with long COVID. They suggested the initiation of institutional campaigns to raise awareness of long COVID among one-stop shops in the healthcare sector and the general population. Additionally, they called for an increase in research activities and government investments regarding the development of treatment structures for long COVID and the expansion of existing therapeutic services. They also wished for the reduction of access barriers to support services and studies, improved communication between treating physicians, and the establishment of one-stop shops for integrated specialist care for people with long COVID. The participants hoped that healthcare providers would accept their long COVID complaints more and provide more emotional support.

### Linking with existing literature

To develop intersectoral coordinated interventions and services for people with long COVID, the literature recommends the design and dissemination of evidence-based multidisciplinary treatment guidelines [9, 26]. In some European countries, such as Austria, France, Germany, or Norway, the first guidelines or action plans have already been published, which are aimed at service providers, medical staff, and/or patients [45, 54, 56, 62]. The development or updating of such care guidelines and plans should increasingly include the perspective of those affected. Currently, the guidelines do not include participatory elements such as the involvement of patient advisory councils [45, 54, 56].

### Strengths and limitations

This study refers to a sample of four focus groups with 23 adults, whose recruitment was limited to a two-month period. In determining the sample size, we followed guidelines to conduct three to five focus groups of five to eight people each [31, 33–35]. Most of the study participants are employed persons of German origin with a residence in the federal state of Lower Saxony. Various population groups (e.g., people with a migration history or older adults) are underrepresented in this study. We excluded participants who could not follow a one-hour conversation from the outset for pragmatic reasons. Therefore, this study does not represent severe cases of illness. Meeting location may have been a barrier for some affected people such as those with mobility impairments. People with long COVID needed access to online platforms (e.g., Instagram) or other respondents to be recruited for this study. A limitation of the data collection method is that in focus groups, individual participants have less speaking time than in one-on-one interviews to provide in-depth insights into their attitudes and experiences. The benefits of focus groups are the lower interviewer or moderator bias and the existing group dynamic effects, which positively influence the commitment and willingness to provide information on the side of the respondents. Due to the collective knowledge base, a focus group can also be more productive than interviews with individuals [31], which was particularly evident in the identification of care barriers and the development of approaches to improve the care situation. In this study, no sex and/or gender analysis was conducted due to the relatively small sample size and especially the underrepresentation of men.

## Conclusions

The study participants mostly reported that they did not receive any support services such as specialist referrals or therapeutic recommendations during a GP consultation due to their long COVID complaints. However, several respondents were satisfied with their GPs who listened, were open, and addressed their problems. Participants described receiving various types of treatments, such as group meetings supported by psychotherapists or individualized occupational therapy treatments. These were majority perceived as helpful. As barriers to accessing such services, participants identified a lack of acceptance of long COVID complaints by their GPs at the micro level, a high level of bureaucracy and long waiting times of several months at specialists and long COVID outpatient clinics at the meso level, and a lack of fundamental care concepts to deal with chronic long COVID ailments in the German healthcare system at the macro level. The following implications for healthcare professionals and policymakers can be derived from the focus groups: (1) GPs should take complaints seriously from the outset, assume a proactive care coordinating role, make timely complaint-oriented specialist referrals, and provide access to specialized points of contact such as long COVID outpatient clinics; (2) treating physicians should offer emotional support to people with long COVID and consider prescribing non-drug therapies, such as group meetings supported by psychotherapists, occupational therapy, or physical therapy; (3) policymakers should initiate measures to raise awareness among one-stop shops in the healthcare sector and healthcare professionals about the complaints and needs of people with long COVID, such as targeted information campaigns; (4) care planners, researchers, and service providers should focus on developing interprofessional evidence-based care and treatment approaches for long COVID; (5) the public sector should promote the expansion of existing care structures such as long COVID outpatient clinics. The overarching goal of healthcare authorities must be to develop consistent, evidence-based, and practice-oriented guidelines for healthcare professionals regarding the diagnosis of long COVID and the medical care and therapeutic treatment of affected patients.

### Electronic supplementary material

Below is the link to the electronic supplementary material.

Supplementary Material 1

## Data Availability

The transcripts generated during this study are not publicly available as they may compromise research participant privacy. It is however available from the corresponding author upon reasonable request within a data-sharing agreement, and subsequent approval from the responsible research ethic boards.

## Abbreviations

COREX: COnsolidated criteria for REporting qualitative research
DEFEAT: DEFEnse Against COVID-19 STudy
GP: General Practitioner
MHH: Hannover Medical School
RKI: Robert Koch-Institute
SARS-CoV-2: Severe Acute Respiratory Syndrome CoronaVirus type 2
UMG: University Medical Center Göttingen
