# Supplementary material for: “There is no one who helps you with it”: experiences of people with long COVID regarding medical care, therapeutic measures, and barriers in the German healthcare system: results of a qualitative study with four focus groups
Source: BMC Health Serv Res. 2023 Oct 26;23:1160. doi: 10.1186/s12913-023-10170-x (PMC10601213; doi:10.1186/s12913-023-10170-x)
Supplement: Supplementary file 1 — Supplementary Material 1 [file 12913_2023_10170_MOESM1_ESM.docx]

Supplementary file

**Moderation guideline**

1st stimulus with question (recorded radio report on the topic of COVID long-term consequences in everyday life and medical support in post COVID (up to minute 02:40 [33])).

**What thoughts do you associate with what you just heard in the report?**

2nd stimulus with question.

**When you think of health limitations such as concentration problems and fatigue - what impact do they have on your working life, your family life and your social contacts?**

3rd stimulus with question.

Statement of an interviewee: "I have to accept it somewhere, but at the moment I feel left alone by the doctors from the point of view that just nothing happens except that I am put on sick leave. And that's a bit like sitting here at home and doing nothing [...]. I say yes, it's good that at least with the doctors, not all of them, but with the doctors where I've been so far, it was mostly accepted and taken seriously, but that they dealt with the issue or said 'come on, we'll do it now. We'll look at something else now, we'll try something or other'. I didn't experience that, not at all. No interest at all in helping in any way."

**What do you think about this?**

4th question.

**Imagine you would live in a world with unlimited resources: What would perfect care and support for people with your health limitations look like there?**

**Optional questions:**

1. When did you become infected with coronavirus?
2. Did you receive a corona vaccination and if so, when was it?
3. How do people in your environment take your health limitations into consideration?
4. How do you evaluate the political measures to protect against the Corona pandemic? In what ways do these measures have an impact on your situation in the context of long COVID or post COVID?
5. What strategies have you found for yourself to cope with your disease and to what extent did you develop strategies to compensate for limitations?
